# Real-world evidence of constipation and laxative use in the Korean population with chronic kidney disease from a common data model

**DOI:** 10.1038/s41598-024-57382-7

**Published:** 2024-03-19

**Authors:** Kipyo Kim, Ji-Eun Kim, Jae Ho Kim, Seong Hee Ahn, Chai Young Jung, Seun Deuk Hwang, Seoung Woo Lee, Joon Ho Song

**Affiliations:** 1 Division of Nephrology and Hypertension, Department of Internal Medicine, Inha University Hospital, Inha University College of Medicine, Incheon, 22332 Republic of Korea; 2 Department of Endocrinology and Metabolism, Inha University Hospital, Inha University College of Medicine, Incheon, 22332 Republic of Korea; 3 Biomedical Research Institute, Inha University Hospital, Incheon, 22332 Republic of Korea

**Keywords:** Kidney diseases, Gastrointestinal diseases, Databases

## Abstract

Constipation is a highly prevalent gastrointestinal disorder in patients with chronic kidney disease (CKD). However, our understanding of its epidemiology and management in CKD is limited. We aimed to explore real-world data on constipation and laxative use in patients with CKD in a nationwide population-based cohort from the Korean Health Insurance Review and Assessment-National Patient Sample database. This study analyzed retrospective health claims data in Korea from 2012 to 2017 that were transformed into the Observational Medical Outcomes Partnership Common Data Model. The pooled proportion of constipation diagnoses was 30.5% in all patients with CKD and 15.9%, 16.5%, 17.4%, 29.9%, and 43.3% in patients with CKD stages 1–5, respectively, suggesting a higher prevalence in advanced CKD. Patients receiving peritoneal dialysis or hemodialysis had the highest prevalence of constipation, while transplant recipients showed a prevalence comparable to that of patients with early CKD. Patients with CKD had a significantly higher risk of constipation than age- and sex-matched non-CKD individuals (range of odds ratio [OR]:1.66–1.90). Laxative prescribing patterns differed by CKD severity. Osmotic agents were prescribed in more than half of patients with advanced CKD, while magnesium salts and bulking agents were prescribed less frequently. The CKD patients with constipation were more likely to be prescribed constipation-inducing medications, including antipsychotic and neurological medications. Our findings provide real-world constipation and laxative prescription status in the Korean CKD population, revealing a significantly higher risk of constipation and different laxative prescribing patterns in patients with CKD.

## Introduction

Constipation is one of the leading gastrointestinal symptoms commonly encountered in the general population, particularly among older adults^1^. Constipation significantly impairs the health-related quality of life (QoL) of affected individuals and poses considerable economic and healthcare burdens^2^. Recent evidence suggests a close association between constipation and clinical outcomes such as cardiovascular disease, chronic kidney disease (CKD) progression, and mortality^3–6^. In particular, numerous observational studies have reported a higher prevalence of constipation in patients with CKD. Although the global prevalence of constipation in the general population has been estimated at approximately 14%, the prevalence in patients with CKD is reported to be much higher^7^. A recent metaanalysis reported a constipation prevalence of 38.8% in patients with advanced non-dialysis CKD^8^. Patients with end-stage kidney disease (ESKD) have been shown to have a substantially higher constipation prevalence, with some studies reporting over 50%^9^. CKD is associated with constipation both clinically and pathophysiologically. Dietary and fluid restrictions, high pill burdens, comorbidities, gut dysbiosis, and uremic milieu may all contribute to chronic constipation^10^.

Despite these observations, most research topics on constipation in patients with CKD remain unexplored. The exact prevalence and incidence rates of constipation in patients with CKD remain undetermined; most studies involved a relatively small number of participants, often less than 100^8^. The detailed characteristics and epidemiological data on constipation in patients with CKD are largely unknown. Furthermore, the clinical impacts and outcomes of constipation in patients with CKD have rarely been investigated. Patients with CKD are often managed for constipation in primary care. However, there are no specific guidelines for managing constipation in patients with CKD. The general guidelines for CKD from the Kidney Disease Improving Global Outcomes do not cover the management of constipation^11^. Given the growing interest in patient-reported outcomes in managing patients with CKD, constipation is a major component of improving the quality of care^12,13^. In addition, as recent studies have highlighted the gut microbiota as a therapeutic target for CKD and gut motility is associated with dysbiosis, constipation management is no longer a negligible issue^14,15^. Generally, CKD-specific cohorts have no or limited data on gastrointestinal symptoms. Most well-designed cohort studies of CKD have no detailed information on constipation or laxative use. To address this knowledge gap, we conducted a nationwide population-based study using the Korean Health Insurance Review and Assessment-National Patient Sample (HIRA-NPS) database. Our data analysis was based on the common data model (CDM), using standardized structures and vocabularies for reliable evidence. We aimed to provide real-world evidence of the epidemiology of constipation and laxative use in patients with CKD.

## Methods

### Data source

We used the HIRA-NPS database, which is a nationwide standard cohort based on claims data from 2012 to 2017. In Korea, national health insurance covers almost 98% of the national population^16^; HIRA-NPS data are representative datasets generated during the reimbursement for healthcare services, sampling approximately 3% of the total population. HIRA-NPS was sampled using a proportionate stratified sampling method by dividing total population into 32 strata (2 gender strata and 16 age strata)^17^. Estimates from HIRA-NPS demonstrates a high level of representativeness for the total population^17^. The Korean Health Insurance Review and Assessment (HIRA) and HIRA-NPS data have enabled diverse real-world data analyses in previous publications^18–21^. HIRA-NPS data from 2012 to 2017 were transformed into the Observational Medical Outcomes Partnership Common Data Model (OMOP CDM, version 5.0) developed by the Observational Health Data Sciences and Informatics (OHDSI) network with standard vocabulary and ontology^22^.

### Study design and cohort definition

Definitions of the concept sets used are presented in Supplementary Table 1. Descendant concepts of each concept set were also used in the analysis. CKD concepts were defined according to CKD stages. The CKD stage was determined by the highest CKD stage code among patient condition occurrences. We only included patients with 2 or more inpatient or outpatient ICD codes for each condition, similar to other studies using HIRA database^23,24^. Patients who underwent kidney transplantation (KT) were investigated separately from the total CKD population. Patients undergoing peritoneal dialysis (PD) and hemodialysis (HD) were identified using procedure-occurrence concepts. Constipation medications included 8 types of laxatives (psyllium, polycarbophil, polyethylene glycol [PEG], magnesium salts, lactulose, lactitol, bisacodyl, and docusate). Some constipation drugs, such as PEG, are often used for bowel preparation for colonoscopy; thus, cases with a drug era length of less than 3 days were excluded. Over-the-counter drugs such as senna and non-benefit drugs were not included in the analysis. This study was conducted in accordance with the World Medical Association Declaration of Helsinki and was approved by the Institutional Review Board of Inha University Hospital (IRB No. 2023–08-034). The requirement for informed consent from the study subjects was waived by the IRB of Inha University Hospital due to the retrospective study design. All CDM-converted data used in the analysis were fully anonymized.

### Statistical analysis

Data analysis was performed using an interactive analysis tool ATLAS (version 2.12.0) provided by the FEEDER-NET platform (https://feedernet.com) ^25^. We used the characterization, cohort pathways, and estimation functions of ATLAS and R (version 4.2.1; R Foundation for Statistical Computing, Vienna, Austria). Fisher’s exact test was used to compare differences in drug prescriptions between CKD patients with and without constipation. The risk of constipation and laxative use was evaluated in patients with CKD compared to age- and sex-matched individuals without CKD using logistic regression. The cohort entry event was the initial occurrence of diagnostic codes, and the time-at-risk was defined as the time between 1 day after cohort start date and cohort end date. Demographic covariates were included in the model fitting, and regularization was applied. KT recipients and patients receiving HD or PD were also compared in the same manner. Patients with stage-unspecified CKD were only included in the analyses where CKD stage and severity were not used. P-values of < 0.05 were considered statistically significant, and the Bonferroni correction was applied for multiple testing.

## Results

### Characteristics of the study population

A total of 39,293 individuals diagnosed with CKD between 2012 and 2017 were included in this study. Table 1 presents the characteristics of the participants. The overall number of patients with CKD increased noticeably from 2012 to 2017. This trend reflects the age distribution of the Korean population and the recent rapid population aging^26^. Recent data on CKD and ESKD populations in Korea also provide consistent findings^27,28^. Patients with CKD stage 1 and 2 were less likely to be captured, possibly due to low rates of screening or follow-up. Patients with CKD stage 5 were more likely to be recruited based on the Korean Classification of Disease (KCD) codes due to a high coverage benefit. Males were 60.1%, and the most common age groups were middle (40–64 years) and old age groups (65–79 years). However, individuals of ≥ 80 years have also increased markedly from 2015. The number of ESKD patients identified using procedure codes also increased over time, mostly attributed to the increasing number of HD patients. Kidney transplantation also increased by 40% during the study period. Patients undergoing PD were reduced from 14.3% to 9.1% of all ESKD patients.

**Table 1:** Characteristics of the study population.

	2012	2013	2014	2015	2016	2017
Total CKD population*	5031	5751	6190	6647	7642	8032
CKD Stage 1	114	125	142	164	178	197
CKD Stage 2	229	282	280	321	429	380
CKD Stage 3	251	316	399	439	488	543
CKD Stage 4	371	462	478	511	610	710
CKD Stage 5^§^	1880	1972	2063	2263	2440	2497
ESKD	1672	1756	1840	2028	2183	2219
HD/PD	1433/239	1498/258	1604/236	1797/231	1961/222	2016/203
Unspecified	2186	2594	2828	2949	3497	3705
Kidney transplantation	564	575	595	674	727	790
Sex, n(%)						
Male	2997 (59.6)	3412 (59.3)	3737 (60.4)	4065 (61.2)	4559 (59.7)	4853 (60.4)
Female	2034 (40.4)	2339 (40.7)	2453 (39.6)	2582 (38.8)	3083 (40.3)	3179 (39.6)
Age groups, n(%)						
0–19 years	18 (0.4)	26 (0.5)	26 (0.4)	22 (0.3)	28 (0.4)	10 (0.1)
20–39 years	302 (6.0)	387 (6.7)	405 (6.5)	318 (4.8)	354 (4.6)	202 (2.5)
40–64 years	2182 (43.4)	2374 (41.3)	2775 (44.8)	2647 (39.8)	2957 (38.7)	2240 (27.9)
65–79 years	2023 (40.2)	2350 (40.9)	2484 (40.1)	2776 (41.8)	3217 (42.1)	3080 (38.3)
≥ 80 years	506 (10.1)	614 (10.7)	500 (8.1)	884 (13.3)	1086 (14.2)	2500 (31.1)
Hypertension, n (%)	2381 (47.3)	2910 (50.6)	3173 (51.3)	3468 (52.2)	4589 (60.0)	4906 (61.1)
Diabetes mellitus, n (%)	2275 (45.2)	2612 (45.4)	2855 (46.1)	3131 (47.1)	3733 (48.8)	3633 (45.2)
CCI, mean ± SD	4.2 ± 2.1	4.2 ± 2.2	4.2 ± 2.1	4.3 ± 2.2	4.4 ± 2.2	4.2 ± 2.1

CKD, chronic kidney disease; ESKD, end-stage kidney disease; HD, hemodialysis; PD, peritoneal dialysis; CCI, Charlson comorbidity index. *Kidney transplantation was not included. ^§^CKD stage 5 included 5ND and 5D.

### Constipation diagnosis and laxative use in CKD

**Figure 1:**
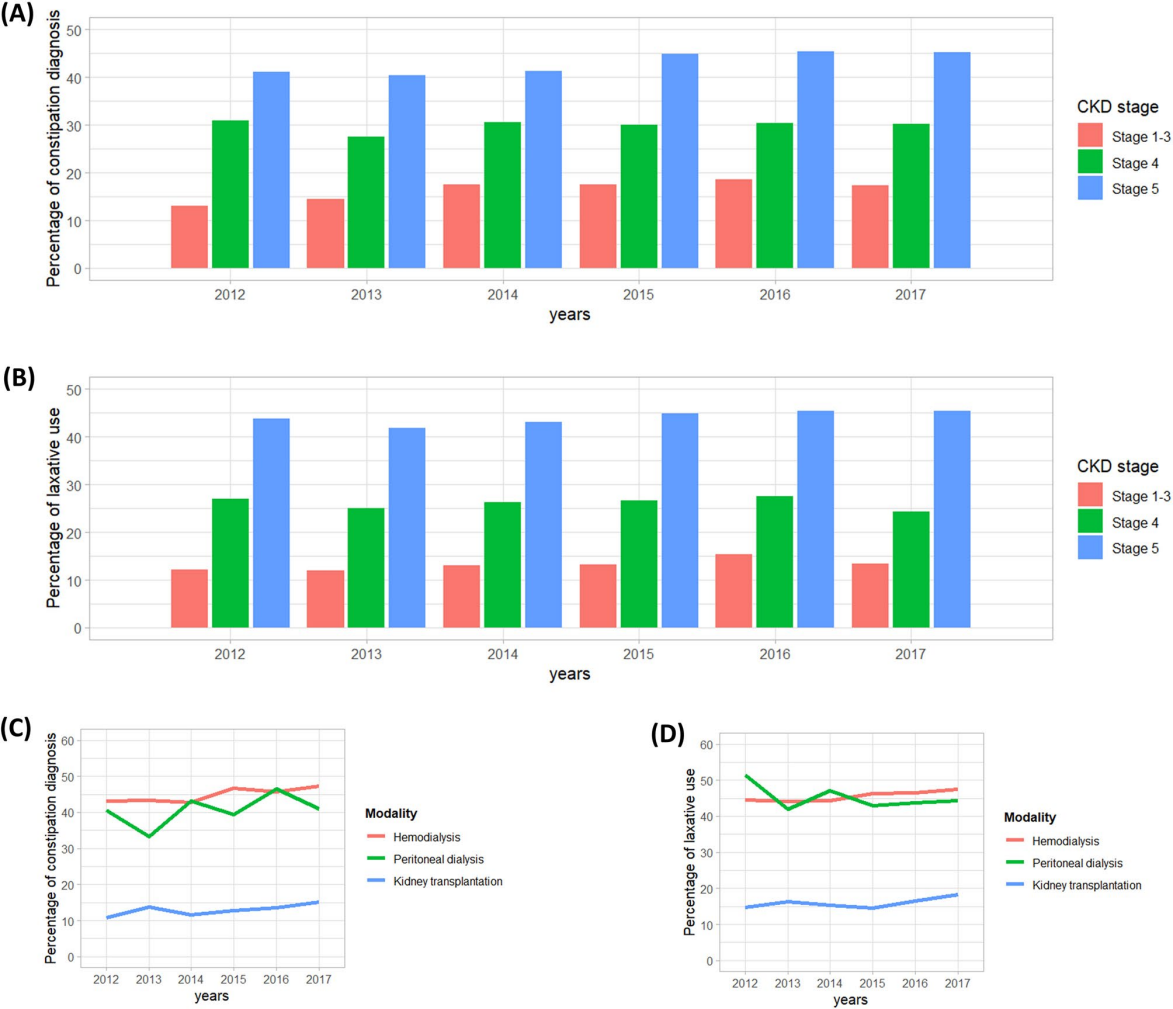
Proportion of constipation and laxative prescriptions in patients with chronic kidney disease (CKD). (**A**) Constipation diagnosis according to the CKD stages. (**B**) Laxative use according to the CKD stages. (**C**) Constipation diagnosis in patients with end-stage kidney disease (ESKD) including kidney transplant (KT) recipients. (**D**) Laxative use in patients with ESKD including KT recipients.

The proportions of constipation and laxative exposure in patients with CKD are shown in Fig. 1. Overall, patients with higher CKD stages were more likely to be diagnosed with constipation and treated with laxatives. The proportions of constipation and laxative use were similar in CKD stages 1 to 3 but markedly increased in CKD stage 4 or higher. The pooled proportion of constipation during the study period was 32.3% in all patients with CKD and 15.9%, 16.5%, 17.4%, 29.9%, and 43.3% in patients with CKD stages 1–5, respectively (16.8% for CKD stages 1 to 3). Similarly, 30.1% of all patients with CKD and 11.7%, 12.9%, 14.5%, 26.1%, and 44.2% of patients with CKD stages 1–5 were prescribed laxatives in the pooled cohort (13.4% for CKD stages 1 to 3). Patients with ESKD had the highest pooled proportions of constipation and laxative use of 44.5% (HD: 45.0%, PD: 40.5%) and 45.7% (HD: 45.7%, PD: 45.2%), respectively. Constipation diagnosis was slightly higher in HD patients compared to PD patients. However, the proportion of laxative prescription was similar between PD and HD patients. (Figs. 1C and 1D). KT recipients showed relatively lower prevalence of constipation and laxative use compared to those on HD/PD (pooled proportion 13.1% and 16.1%). In the logistic regression analysis, patients with CKD showed significantly higher odds ratios (ORs) for constipation and laxative use than age- and sex-matched non-CKD cohorts (Table 2). The estimated ORs for constipation and laxative use ranged from 1.66 to 1.90 and 2.18 to 2.33, respectively. Patients with advanced CKD had a significantly higher risk of constipation and laxative use compared to age- and sex-matched patients with mild CKD (Supplementary Table 2). In addition, patients receiving HD or PD had significantly higher ORs for constipation and laxative use compared to age- and sex-matched KT recipients (Supplementary Table 3 and 4).

**Table 2:** Risks of constipation diagnosis and laxative use in patients with CKD.

Years		Constipation		Laxative use	
		Non-CKD	CKD	Non-CKD	CKD
2012	Event n/total n	511/4002	886/4002	408/4091	963/4091
	OR (95% CI)	Reference	1.66 (1.34–2.05)	Reference	2.26 (1.81–2.82)
2013	Event n/total n	585/4573	940/4573	470/4747	1046/4747
	OR (95% CI)	Reference	1.68 (1.48–1.90)	Reference	2.32 (2.04–2.63)
2014	Event n/total n	597/4907	1096/4907	486/5103	1163/5103
	OR (95% CI)	Reference	1.81 (1.27–2.57)	Reference	2.33 (1.63–3.32)
2015	Event n/total n	703/5212	1229/5212	589/5456	1307/5456
	OR (95% CI)	Reference	1.74 (1.53–1.98)	Reference	2.23 (1.96–2.53)
2016	Event n/total n	774/6015	1416/6015	670/6246	1459/6246
	OR (95% CI)	Reference	1.90 (1.50–2.41)	Reference	2.18 (1.72–2.78)
2017	Event n/total n	835/6297	1488/6297	707/6539	1551/6539
	OR (95% CI)	Reference	1.73 (1.43–2.10)	Reference	2.26 (1.86–2.76)

All odd ratios were adjusted for age group, sex, and condition occurrence at any prior time. Patients with stage-unspecified CKD were included in the analysis. The non-CKD and CKD groups were matched according to age groups and sex. CKD, chronic kidney disease; OR, odds ratio; CI, confidence interval.

### Treatment pathways for laxative prescriptions

**Figure 2:**
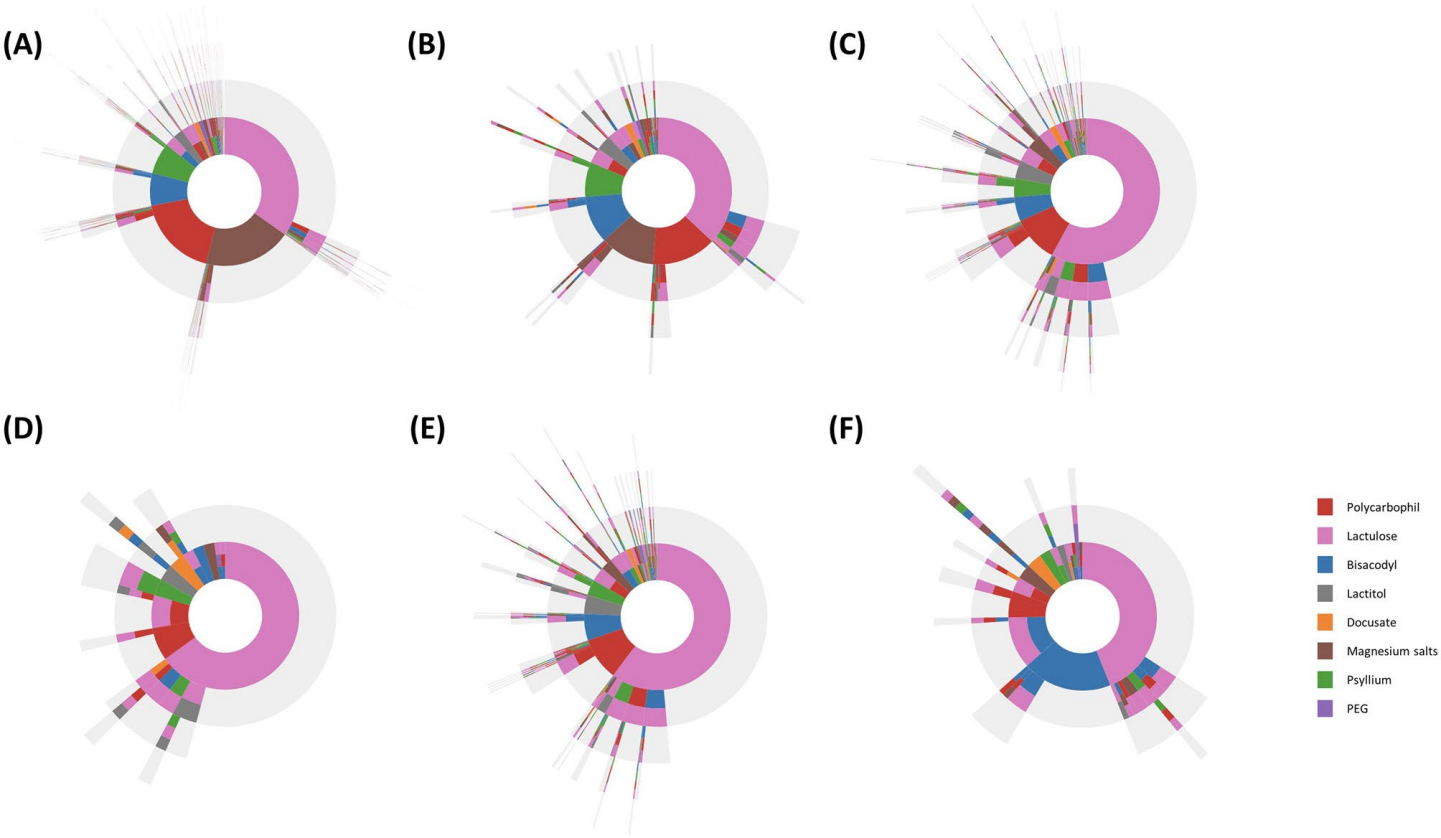
Treatment pathways for laxatives in patients with (**A**) non-chronic kidney disease (non-CKD), (**B**) mild chronic kidney disease (CKD) (CKD stages 1–3), (**C**) advanced CKD (CKD stages 4–5), (**D**) peritoneal dialysis (PD), (**E**) hemodialysis (HD), and (**F**) kidney transplantation. Sunburst plots are drawn using data in 2017. Plots using data in 2012–2016 are presented in Supplementary Fig. 1–5. PEG = polyethylene glycol.

The treatment pathway of laxative prescriptions was examined in the different subgroups (Fig. 2 and Supplementary Fig. 1–5). In individuals without CKD, the commonly prescribed laxatives are lactulose, magnesium salts, and polycarbophil. However, as CKD progressed from the mild to advanced stages, the use of magnesium salts and polycarbophil decreased markedly, while the use of lactulose gradually increased. In a sunburst plot, the outer rings represent the second-line and subsequent therapies in individual patients. Sunburst plots of patients with CKD had a larger second ring area, indicating a greater need for second-line laxatives. In patients with ESKD, lactulose accounts for over 60% of all laxatives, followed by polycarbophil. Magnesium salts are rarely used in patients with ESKD. Sunburst plots of patients on HD showed longer spikes in the third and fourth outer rings, suggesting more frequent changes in the laxatives. Most of the second- or higher-line therapies are osmotic agents, such as lactulose and lactitol. KT recipients were prescribed mainly lactulose and bisacodyl, but the proportion of lactulose was similar to patients with mild CKD.

### Differences in drug prescriptions

We evaluated differences in medication prescriptions between CKD patients with and without constipation. Excluding laxatives, the top 10 drug groups sorted by ORs are shown in Table 3 (individual drugs are presented in Supplementary Table 5). The CKD patients with constipation were more frequently prescribed medications known to cause constipation, such as antipsychotics (risperidone, quetiapine, and haloperidol), antidepressants (nortriptyline, amitriptyline, and escitalopram), antiepileptics (valproate and clonazepam), hypnotics and sedatives (zolpidem), anxiolytics (diazepam and lorazepam), opioids (fentanyl, oxycodone, and tramadol), nonsteroidal anti-inflammatory drugs (celecoxib and diclofenac), and antimuscarinics (solifenacin and propiverine)^29^. Of note, the findings suggest that drug categories such as antipsychotics may pose the greatest risk for constipation in patients with CKD, and that special caution is needed when prescribing these drugs. In addition, drugs commonly used in patients with CKD have been identified, such as diuretics (furosemide and spironolactone), potassium binders (calcium polystyrene sulfonate), and iron supplements (ferrous sulfate). While most medications with high ORs may be directly related to constipation from their adverse effects, others are possibly linked to specific medical conditions, such as hypokalemia (potassium chloride) and poor oral intake (megestrol).

**Table 3:** Drug groups prescribed significantly more often in CKD patients with constipation.

2012		2013		2014		2015		2016		2017	
Drug groups	OR (95% CI)	Drug groups	OR (95% CI)	Drug groups	OR (95% CI)	Drug groups	OR (95% CI)	Drug groups	OR (95% CI)	Drug groups	OR (95% CI)
Antipsychotics	3.18 (2.29–4.43)	Antipsychotics	2.80 (2.06–3.81)	Antipsychotics	2.77 (2.05–3.75)	Antipsychotics	3.21 (2.37–4.36)	Antipsychotics	3.04 (2.34–3.97)	Cough suppressants	2.51 (1.73–3.65)
Antiparkinson drugs	2.37 (1.67–3.39)	Hypnotics and sedatives	2.56 (2.14–3.05)	Hypnotics and sedatives	2.23 (1.87–2.65)	Antiparkinson drugs	2.18 (1.58–3.02)	Antiepileptics	2.21 (1.87–2.61)	Antipsychotics	2.22 (1.76–2.79)
Antiepileptics	2.15 (1.74–2.67)	Cough suppressants	2.34 (1.57–3.51)	Antidepressants	2.14 (1.80–2.53)	Antiepileptics	2.09 (1.75–2.50)	Hypnotics and sedatives	2.10 (1.78–2.46)	Hypnotics and sedatives	1.93 (1.66–2.25)
Hypnotics and sedatives	1.96 (1.62–2.37)	Antidepressants	2.18 (1.82–2.60)	Anti-dementia drugs	2.12 (1.60–2.79)	Hypnotics and sedatives	1.96 (1.65–2.33)	Loop diuretics	1.95 (1.74–2.20)	Antiepileptics	1.88 (1.59–2.21)
Potassium-sparing agents	1.85 (1.41–2.42)	Drugs used in BPH	2.13 (1.78–2.56)	Antiepileptics	1.93 (1.60–2.33)	Loop diuretics	1.95 (1.72–2.21)	Antidepressants	1.93 (1.65–2.26)	Loop diuretics	1.82 (1.62–2.04)
Antidepressants	1.84 (1.53–2.21)	Antiepileptics	2.07 (1.68–2.55)	Drugs used in BPH	1.93 (1.63–2.29)	Drugs used in BPH	1.87 (1.59–2.19)	Drugs used in BPH	1.89 (1.62–2.20)	Anti-dementia drugs	1.71 (1.38–2.11)
Drugs for acid-related disorders	1.80 (1.59–2.04)	Anxiolytics	1.90 (1.64–2.20)	Anxiolytics	1.90 (1.64–2.19)	Anti-dementia drugs	1.87 (1.43–2.43)	Vasodilators	1.77 (1.49–2.12)	Anxiolytics	1.65 (1.44–1.88)
Drugs used in BPH	1.76 (1.45–2.14)	Vasodilators	1.82 (1.48–2.24)	Potassium-sparing agents	1.84 (1.43–2.37)	Antidepressants	1.77 (1.49–2.10)	Anti-dementia drugs	1.75 (1.40–2.17)	Drugs used in BPH	1.60 (1.39–1.85)
Loop diuretics	1.70 (1.48–1.95)	Opioids	1.75 (1.55–1.97)	Drugs for acid-related disorders	1.82 (1.63–2.03)	Anxiolytics	1.74 (1.52–2.01)	Anxiolytics	1.74 (1.52–1.99)	Vasodilators	1.58 (1.31–1.89)
Opioids	1.55 (1.36–1.76)	Loop diuretics	1.70 (1.48–1.94)	Loop diuretics	1.77 (1.55–2.01)	Drugs for acid-related disorders	1.70 (1.53–1.89)	Drugs for acid-related disorders	1.72 (1.56–1.90)	Antidepressants	1.48 (1.27–1.73)

Laxatives were excluded. Potassium-sparing agents include spironolactone and amiloride. Drugs for acid-related disorders include histamine-2 receptor antagonists and proton pump inhibitors. Vasodilators include nicorandil, molsidomine, glyceryl trinitrate, isosorbide mononitrate, and isosorbide dinitrate. BPH, benign prostatic hypertrophy; OR, odds ratio; CI, confidence interval.

## Discussion

In this study, we present real-world data on constipation and laxative use in patients with CKD in a nationwide cohort based on health insurance claims data. Consistent with previous observations, our data show a high prevalence of constipation in patients with CKD. In particular, constipation and laxative prescriptions were notably higher in the later stages of CKD, with ESKD being the highest. The risks of constipation and laxative use in patients with CKD were also significantly higher than those in age- and sex-matched populations without CKD. Second-line laxative use increases with the progression of CKD. The most frequently prescribed laxative is the osmotic agent lactulose, which accounts for nearly half of all laxative prescriptions for patients with advanced CKD. We also found differences in the prescription patterns of constipation-inducing drugs, including antipsychotic and neurological medications, between CKD patients with and without constipation.

While previous studies on constipation have mostly focused on patients at specific stages of CKD, such as ESKD, this study explored constipation and laxative use across all stages of CKD in a population-based cohort, which is a major strength of our study. The prevalence of constipation largely depends on the diagnostic tools used. Early studies used a self-reported definition of constipation; however, recent studies have favored the more standardized Rome criteria based on defecation symptoms and stool forms. In general, the prevalence based on the Rome criteria was lower than the patient-reported prevalence. To date, only a small number of studies have examined the prevalence of constipation in patients with non-dialysis CKD. A recent meta-analysis reported the pooled prevalence of self-reported and functional constipation in different CKD stages^8^. The prevalence of self-reported and functional constipation in patients with CKD stage 4–5 was 38.78% and 21.97%, respectively. Therefore, the prevalence of 29.9% (CKD stage 4), and 43.3% (CKD stage 5) in this study was close to the self-reported prevalence. The self-reported prevalence of constipation in CKD stage 3 was 29.75% in metaanalysis, which was mainly driven by two recent studies^30,31^. However, these studies only included patients with CKD stage 3B (GFR 30–44 ml/min/1.73 m^2^), while we included all stage 3 patients, resulting in a relatively low prevalence of 17.4%. For patients with CKD stages 1 and 2, small studies with sample sizes of 22 or less have reported a prevalence of 12.5–31.8%^32,33^. In our study, the estimated prevalence of constipation in CKD stages 1 and 2 was 15.9% and 16.5%, respectively, which was much lower than in advanced CKD.

On the other hand, numerous reports on constipation in patients with CKD have mainly focused on ESKD populations, which show a significantly higher prevalence. The prevalence of constipation has been reported to be higher in HD patients than in PD patients, which may be due to fluid restriction during the interdialytic period and low physical activity^34^. A study by Yasuda et al. compared the prevalence of constipation between patients on PD (n = 204) and HD (n = 268) and found a prevalence defined using a questionnaire of 28.9% in PD patients and 63.1% in HD patients^35^. Studies using the Rome criteria and with a sample size of 100 or more have reported a prevalence of 30.5–71.7% in HD patients and 14.2% in PD patients^36–38^. In this study, the pooled prevalence of constipation in HD and PD patients was estimated to be 45.0% and 40.5%, respectively. We found that constipation was more prevalent in patients on HD than those on PD, but the difference was not as pronounced as in previous studies. Notably, patients receiving PD had a higher proportion of laxative use than expected from their constipation diagnosis. This may indicate chronic use of laxatives in patients on PD regardless of the severity of their symptoms. Indeed, constipation can cause catheter dislocation, leading to drainage failure, which can lead clinicians to prescribe more laxatives. Of note, KT recipients showed a substantially lower prevalence of constipation and laxative use compared to patients with advanced CKD or ESKD. The prevalence of self-reported constipation in KT recipients is reported to be 37–43% in recent questionnaire-based studies^39,40^. However, in contrast to patients with ESKD, diarrhea rather than constipation is the most common and problematic gastrointestinal symptom in KT recipients^40^. These differences are likely related to immunosuppressive agents such as mycophenolate mofetil, low pill burden, normalization of uremic milieu, and changes in the gut microbiota after KT^41^.

We found that lactulose was the most frequently prescribed laxative in Korean patients with CKD. Lactulose is a relatively safe laxative, even in patients with advanced CKD. Also, there is some evidence that lactulose has a beneficial effect on gut microbiota in CKD, suppressing uremic toxins^42,43^. The use of magnesium salts and polycarbophil were greatly reduced in patients with advanced CKD. Magnesium salts act as osmotic agents but are avoided due to the risk of hypermagnesemia in patients with CKD^44^. Half of the patients on PD taking a polycarbophil as first-line therapy switched to the osmotic agent lactulose. Polycarbophil is a bulk-forming agent that can induce abdominal fullness and have a relatively weaker efficacy than osmotic agents. Moreover, polycarbophil is typically administered in the form of a calcium salt, which produces free calcium ions, posing a burden on calcium pools in patients with CKD^45^. It remains uncertain which laxatives are more beneficial for patient outcomes. To date, no direct comparison of mortality between laxatives in patients with CKD has been reported. This topic deserves to be explored further in future well-designed prospective studies. In addition, there is no consensus regarding the management of constipation in patients with CKD. The KDIGO guidelines do not address the management of constipation in CKD^11^, and a recent draft of the KDIGO clinical practice guideline only mentions the role of constipation in terms of hyperkalemia treatment. Recently published algorithms for constipation treatment in patients on HD recommend to avoid magnesium- or phosphate-containing laxatives^46^. The authors of the algorithm suggested nonpharmacological therapies such as fiber, fluid intake, and exercise, followed by pharmacological therapy using PEG, lactulose, senna, and bisacodyl^46^.

Constipation in patients with CKD is speculated to be multifactorial^9^. Fluid and dietary restrictions are among the main causes of constipation. Decreased physical activity and medications commonly used in CKD also affect bowel movements. As expected, our study showed that CKD patients with constipation were frequently prescribed constipation-inducing medications related to their CKD condition, including diuretics, potassium binders, iron supplements, antihistamines for pruritus, and analgesics for chronic pain. However, we also found the greatest risk of constipation in CKD patients who are prescribed psychotropic drugs such as antipsychotics, antiepileptics, and antidepressants. Psychotropic drugs and medications for neurologic disorders such as Parkinson's disease and dementia are known to cause constipation, but the risk of constipation when prescribing these medications in patients with CKD has been relatively unrecognized^47,48^. Therefore, our data suggest that clinicians should be aware of the potential adverse effects of these drug categories in managing patients with CKD. Constipation could also be attributed to multiple comorbidities that are highly prevalent in CKD, such as diabetes, cardiovascular disease, depression, and insomnia, which is consistent with our findings^49,50^. Prior to pharmacotherapy, identifying medications and conditions associated with constipation should be prioritized. In addition, several studies have supported that uremic toxins and alterations in the gut microbiome are the main mechanisms responsible for constipation in CKD, but the causal role and clinical implications of dysbiosis remains elusive^51–53^.

Our study has some limitations. We used retrospective data obtained from the health insurance reimbursement process. We determined CKD based on the diagnostic codes of treated patients, not on GFR or albuminuria. There may be some discrepancies between the HIRA data and actual patient conditions. Therefore, we only included patients with 2 or more inpatient or outpatient ICD codes for CKD per year to reduce false positive cases. Given that patients with early-stage CKD have low health care utilization rates, this method could be particularly effective for selecting true mild CKD patients. On the other hand, we were able to identify nearly all ESKD patients with insurance claim codes because all patients receiving HD or PD in Korea are registered with the differential co-payment system of the National Health Insurance Service^54^. The definition of constipation is also based on diagnostic codes and not clinical diagnostic criteria. Therefore, the estimated prevalence may be higher than that estimated using the Rome criteria. In addition, because data after 2017 were not available, newly developed drugs for constipation, such as serotoninergic agonists and chloride channel activators, were not included. To overcome these limitations, more controlled prospective studies, including recently developed drugs, are needed. Nevertheless, HIRA contains enormous amounts of data, including demographics, diagnoses, prescriptions, and outcomes, extracted from almost the entire Korean population. The findings are not specific to any one region or institution but rather reflect the overall treatment patterns and characteristics of constipation in patients with CKD. To our knowledge, this is the first study to investigate the prevalence of constipation in Korean patients with CKD, including pre-dialysis patients. Therefore, our findings add to the understanding of the epidemiology of constipation in patients with CKD and could serve as a useful reference for future studies.

In conclusion, this study provides real-world evidence on the prevalence of constipation and laxative use according to the CKD stage in a nationwide population-based cohort. We found a significantly higher risk of constipation in patients with CKD than in non-CKD individuals and different prescribing patterns of laxatives.

### Supplementary Information

Supplementary Information.

## Data Availability

The data generated in this study are included in the published article and Supplementary Information files. Raw data were obtained with the approval of the Health Insurance Review and Assessment Service in Korea and are not publicly available.
