# Supplementary material for: Real-world evidence of constipation and laxative use in the Korean population with chronic kidney disease from a common data model
Source: Sci Rep. 2024 Mar 19;14:6610. doi: 10.1038/s41598-024-57382-7 (PMC10951406; doi:10.1038/s41598-024-57382-7)
Supplement: Supplementary file 1 — Supplementary Information. [file 41598_2024_57382_MOESM1_ESM.docx]

Real-world evidence of constipation and laxative use in the Korean population with chronic kidney disease from a common data model

Kipyo Kim^1†^, Ji-Eun Kim^1†^, Jae Ho Kim^1^, Seong Hee Ahn^2^, Chai Young Jung^3^, Seun Deuk Hwang^1^, Seoung Woo Lee^1^, Joon Ho Song^1^*

^1^Division of Nephrology and Hypertension, Department of Internal Medicine, Inha University Hospital, Inha University College of Medicine, Incheon, Republic of Korea.

^2^Department of Endocrinology and Metabolism, Inha University Hospital, Inha University College of Medicine, Incheon, Republic of Korea.

^3^Biomedical Research Institute, Inha University Hospital, Incheon, Republic of Korea.

^†^The first two authors contributed equally to this work.

***Corresponding Author:**

Joon Ho Song, MD, PhD

Division of Nephrology and Hypertension, Department of Internal Medicine, Inha University Hospital, Inha University College of Medicine

27 Inhangro, Jung-gu, Incheon 22332, South Korea

E-mail: jhsong@inha.ac.kr

Tel: +82-32-890-2229

Fax: +82.32-890-2530

**Supplementary information**

Supplementary Table 1. Definitions of the concept sets used in the study.

| Concept sets | Included concept Id | Included concept code | Included name |
| --- | --- | --- | --- |
| Chronic kidney disease | 46271022, 37080795, 443614, 443601,443597, 443612, 443611 | 709044004, 10064848, 431855005, 431856006,433144002, 431857002, 433146000 | Chronic kidney disease, Chronic kidney disease, Chronic kidney disease stage 1, Chronic kidney disease stage 2, Chronic kidney disease stage 3, Chronic kidney disease stage 4, Chronic kidney disease stage 5 |
| Chronic kidney disease stage 1 | 443614 | 431855005 | Chronic kidney disease stage 1 |
| Chronic kidney disease stage 2 | 443601 | 431856006 | Chronic kidney disease stage 2 |
| Chronic kidney disease stage 3 | 443597 | 433144002 | Chronic kidney disease stage 3 |
| Chronic kidney disease stage 4 | 443612 | 431857002 | Chronic kidney disease stage 4 |
| Chronic kidney disease stage 5 | 443611 | 433146000 | Chronic kidney disease stage 5 |
| End-stage kidney disease | 4197217, 4156957, 4080169, 37521163, 4120120, 4108838, 46271816, 4324124, 37521167, 4325212, 4235076 | 79827002, 271418008, 238318009, 10018875, 302497006, 180272001, 710071003, 71192002, 10034660, 428118009, 438342006 | Arteriovenous anastomosis for renal dialysis, Chronic ambulatory peritoneal dialysis catheter procedure, Continuous ambulatory peritoneal dialysis, Haemodialysis, Hemodialysis, Insertion of chronic ambulatory peritoneal dialysis catheter, Management of peritoneal dialysis, Peritoneal dialysis, Peritoneal dialysis, Procedure involving peritoneal dialysis catheter, Replacement of peritoneal dialysis catheter |
| Kidney transplantation | 42539502 | 737295003 | Transplanted kidney present |
| Hemodialysis | 4026915, 4120120, 37521163, 4197217 | 225892009, 302497006, 10018875, 79827002 | Revision of arteriovenous shunt for renal dialysis, Hemodialysis, Haemodialysis, Arteriovenous anastomosis for renal dialysis |
| Peritoneal dialysis | 4235076, 4325212, 37521167, 4324124, 46271816, 4108838, 4080169, 4156957 | 438342006, 428118009, 10034660, 71192002, 710071003, 180272001, 238318009, 271418008 | Replacement of peritoneal dialysis catheter, Procedure involving peritoneal dialysis catheter, Peritoneal dialysis, Peritoneal dialysis, Management of peritoneal dialysis, Insertion of chronic ambulatory peritoneal dialysis catheter, Continuous ambulatory peritoneal dialysis, Chronic ambulatory peritoneal dialysis catheter procedure |
| Constipation | 75860, 35708100 | 14760008, 10010774 | Constipation, Constipation |
| Psyllium | 957797, 19132965, 21600555 | 8928, 82088, A06AC01 | Psyllium, Plantago Seed, ispaghula (psylla seeds) |
| Polycarbophil | 19074641, 940426 | 308934, 20063 | calcium polycarbophil 625 MG Oral Tablet, calcium polycarbophil |
| Polyethylene glycol | 948515, 42899937, 986417, 21600575 | 8516, 1310594, 221147, A06AD15 | Polyethylene Glycols, polyethylene glycol 4000, POLYETHYLENE GLYCOL 3350, macrogol |
| Magnesium salts | 40062408, 993690, 993740, 993631 | 372692, 311429, 332148, 6582 | Magnesium Oxide Oral Tablet, Magnesium Oxide 250 MG Oral Tablet, Magnesium Oxide 250 MG, Magnesium Oxide |
| Lactulose | 42925056, 19003454, 987245 | OMOP4651888, 104148, 6218 | Lactulose 670 MG/ML Oral Suspension, Lactulose 670 MG/ML Oral Solution, Lactulose |
| Lactitol | 19071236 | 28395 | lactitol |
| Bisacodyl | 19020131, 924939 | 198509, 1596 | Bisacodyl 10 MG Rectal Suppository, Bisacodyl |
| Docusate | 21600535, 941258 | A06AA02, 82003 | docusate sodium, Docusate |

Supplementary Table 2. Risks of laxative use and constipation diagnosis according to the severity of CKD.

| **Years** | | **Laxative use** | | **Constipation** | |
| --- | --- | --- | --- | --- | --- |
|  |  | Mild CKD | Advanced CKD | Mild CKD | Advanced CKD |
| 2012 | Event n/total n | 94/928 | 240/928 | 95/913 | 221/913 |
|  | OR (95% CI) | Reference | 2.43 (1.83–3.22) | Reference | 2.16 (1.66–2.83) |
| 2013 | Event n/total n | 139/1121 | 276/1121 | 133/1102 | 250/1102 |
|  | OR (95% CI) | Reference | 1.97 (1.33–2.94) | Reference | 2.13 (1.41–3.23) |
| 2014 | Event n/total n | 116/1198 | 339/1198 | 143/1174 | 294/1174 |
|  | OR (95% CI) | Reference | 2.81 (1.90–4.15) | Reference | 1.93 (1.32–2.83) |
| 2015 | Event n/total n | 155/1388 | 368/1388 | 153/1321 | 325/1321 |
|  | OR (95% CI) | Reference | 2.26 (1.49–3.43) | Reference | 2.12 (1.39–3.24) |
| 2016 | Event n/total n | 188/1537 | 438/1537 | 204/1495 | 397/1495 |
|  | OR (95% CI) | Reference | 2.16 (1.57–2.96) | Reference | 1.88 (1.37–2.60) |
| 2017 | Event n/total n | 181/1657 | 487/1657 | 202/1615 | 448/1615 |
|  | OR (95% CI) | Reference | 2.38 (1.61–3.51) | Reference | 1.98 (1.35–2.90) |

OR, odds ratio; CI, confidence interval.

Supplementary Table 3. Risks of laxative use and constipation diagnosis in kidney transplantation and hemodialysis.

| **Years** | | **Constipation** | | **Laxative use** | |
| --- | --- | --- | --- | --- | --- |
|  |  | Kidney transplantation | Hemodialysis | Kidney transplantation | Hemodialysis |
| 2012 | Event n/total n | 37/529 | 132/529 | 43/529 | 134/529 |
|  | OR (95% CI) | Reference | 3.20 (2.19-4.69) | Reference | 2.80 (1.94-4.04) |
| 2013 | Event n/total n | 44/556 | 118/556 | 49/556 | 131/556 |
|  | OR (95% CI) | Reference | 2.36 (1.65-3.39) | Reference | 2.35 (1.67-3.30) |
| 2014 | Event n/total n | 40/583 | 136/583 | 58/583 | 165/583 |
|  | OR (95% CI) | Reference | 3.18 (2.22-4.57) | Reference | 2.66 (1.95-3.63) |
| 2015 | Event n/total n | 51/670 | 160/670 | 55/670 | 188/670 |
|  | OR (95% CI) | Reference | 2.75 (1.99-3.82) | Reference | 3.00 (2.19-4.11) |
| 2016 | Event n/total n | 59/711 | 199/711 | 63/711 | 212/711 |
|  | OR (95% CI) | Reference | 3.14 (2.32-4.24) | Reference | 3.13 (2.34-4.19) |
| 2017 | Event n/total n | 64/757 | 231/757 | 73/757 | 241/757 |
|  | OR (95% CI) | Reference | 3.42 (2.56-4.57) | Reference | 3.13 (2.39-4.11) |

OR, odds ratio; CI, confidence interval.

Supplementary Table 4. Risks of laxative use and constipation diagnosis in kidney transplantation and peritoneal dialysis.

| **Years** | | **Constipation** | | **Laxative use** | |
| --- | --- | --- | --- | --- | --- |
|  |  | Kidney transplantation | Peritoneal dialysis | Kidney transplantation | Peritoneal dialysis |
| 2012 | Event n/total n | 22/226 | 58/226 | 32/226 | 67/226 |
|  | OR (95% CI) | Reference | 2.64 (1.64-4.40) | Reference | 2.09 (1.39-3.23) |
| 2013 | Event n/total n | 26/237 | 46/237 | 26/237 | 68/237 |
|  | OR (95% CI) | Reference | 1.77 (1.10-2.90) | Reference | 2.62 (1.69-4.18) |
| 2014 | Event n/total n | 17/214 | 63/214 | 22/214 | 62/214 |
|  | OR (95% CI) | Reference | 3.71 (2.22-6.53) | Reference | 2.82 (1.76-4.68) |
| 2015 | Event n/total n | 15/217 | 49/217 | 17/217 | 51/217 |
|  | OR (95% CI) | Reference | 3.27 (1.88-6.03) | Reference | 3.00 (1.77-5.35) |
| 2016 | Event n/total n | 19/207 | 59/207 | 20/207 | 54/207 |
|  | OR (95% CI) | Reference | 3.11 (1.89-5.35) | Reference | 2.70 (1.64-4.62) |
| 2017 | Event n/total n | 21/203 | 53/203 | 22/203 | 59/203 |
|  | OR (95% CI) | Reference | 2.52 (1.55-4.27) | Reference | 2.68 (1.67-4.47) |

OR, odds ratio; CI, confidence interval.

Supplementary Table 5. Individual drugs prescribed significantly more often in CKD patients with constipation.

| 2012 | | 2013 | | 2014 | |
| --- | --- | --- | --- | --- | --- |
| Medications | OR (95% CI) | Medications | OR (95% CI) | Medications | OR (95% CI) |
| Risperidone | 5.51 (2.32-14.5) | Risperidone | 4.12 (2.11-8.35) | Haloperidol | 3.96 (2.12-7.65) |
| Haloperidol | 3.64 (1.92-7.11) | Propiverine | 3.78 (2.10-6.97) | Risperidone | 3.90 (2.00-7.91) |
| Levodopa | 3.51 (1.84-6.88) | Nortriptyline | 3.30 (1.73-6.40) | Bethanechol | 3.72 (2.19-6.46) |
| Labetalol | 3.37 (1.93-6.00) | Triazolam | 3.27 (2.08-5.17) | Oxycodone | 3.36 (1.90-6.04) |
| Quetiapine | 3.23 (1.99-5.30) | Zolpidem | 3.15 (2.46-4.04) | Solifenacin | 2.88 (1.80-4.66) |
| Fentanyl | 3.17 (1.98-5.13) | Fentanyl | 2.88 (2.03-4.10) | Propiverine | 2.73 (1.66-4.55) |
| Terazosin | 2.53 (1.64-3.90) | Escitalopram | 2.84 (1.81-4.50) | Escitalopram | 2.73 (1.80-4.17) |
| Dimenhydrinate | 2.50 (1.83-3.43) | Quetiapine | 2.82 (1.86-4.30) | Fentanyl | 2.63 (1.72-4.03) |
| Nebivolol | 2.31 (1.54-3.47) | Lorazepam | 2.72 (1.91-3.86) | Lorazepam | 2.57 (1.85-3.59) |
| Gabapentin | 2.28 (1.67-3.11) | Tianeptine | 2.71 (1.72-4.28) | Quetiapine | 2.56 (1.74-3.78) |
| Zolpidem | 2.21 (1.69-2.87) | Isosorbide | 2.63 (1.69-4.09) | Trazodone | 2.49 (1.67-3.71) |
| Potassium chloride | 2.08 (1.72-2.52) | Clonazepam | 2.62 (1.62-4.26) | Zolpidem | 2.37 (1.89-2.98) |
| Sodium bicarbonate | 2.02 (1.76-2.32) | Codeine | 2.46 (1.57-3.88) | Amitriptyline | 2.36 (1.79-3.12) |
| Amitriptyline | 1.90 (1.44-2.49) | Terazosin | 2.40 (1.55-3.71) | Omeprazole | 2.27 (1.47-3.51) |
| Spironolactone | 1.85 (1.41-2.43) | Ketorolac | 2.30 (1.53-3.45) | Alprazolam | 2.24 (1.76-2.85) |
| Tamsulosin | 1.85 (1.44-2.37) | Tamsulosin | 2.30 (1.82-2.89) | Potassium chloride | 2.21 (1.83-2.67) |
| Ranitidine | 1.82 (1.56-2.13) | Sodium bicarbonate | 2.25 (1.96-2.57) | Donepezil | 2.10 (1.54-2.87) |
| Furosemide | 1.76 (1.52-2.04) | Potassium chloride | 2.18 (1.81-2.63) | Trimetazidine | 2.07 (1.53-2.79) |
| CPS | 1.74 (1.46-2.07) | Alprazolam | 2.09 (1.63-2.67) | Tamsulosin | 2.04 (1.65-2.52) |
| Famotidine | 1.71 (1.30-2.24) | Famotidine | 2.06 (1.57-2.70) | Pregabalin | 2.03 (1.45-2.82) |
| Sucralfate | 1.70 (1.37-2.10) | Amitriptyline | 2.04 (1.54-2.69) | Isosorbide dinitrate | 1.95 (1.38-2.76) |
| Tramadol | 1.68 (1.45-1.94) | Gabapentin | 1.92 (1.39-2.63) | Sodium bicarbonate | 1.95 (1.72-2.22) |
| Bismuth oxide | 1.63 (1.27-2.08) | Nicorandil | 1.88 (1.37-2.57) | CPS | 1.90 (1.62-2.22) |
| Ferrous Sulfate | 1.57 (1.33-1.84) | Dimenhydrinate | 1.87 (1.37-2.53) | Dimenhydrinate | 1.88 (1.41-2.51) |
| Clopidogrel | 1.56 (1.28-1.90) | Diazepam | 1.87 (1.49-2.33) | Gabapentin | 1.86 (1.38-2.48) |
| Diclofenac | 1.48 (1.22-1.81) | Tramadol | 1.84 (1.61-2.11) | Clopidogrel | 1.85 (1.55-2.21) |
| Acetaminophen | 1.37 (1.20-1.57) | Diphenylpyraline | 1.84 (1.43-2.36) | Furosemide | 1.85 (1.61-2.12) |
| Chlorpheniramine | 1.33 (1.16-1.53) | Hydroxyzine | 1.80 (1.36-2.38) | Cilostazol | 1.84 (1.36-2.48) |
|  |  | Trimebutine | 1.77 (1.45-2.15) | Pantoprazole | 1.82 (1.40-2.37) |
|  |  | Furosemide | 1.76 (1.53-2.03) | Spironolactone | 1.82 (1.41-2.34) |
|  |  | Pantoprazole | 1.76 (1.32-2.33) | Hydroxyzine | 1.79 (1.36-2.35) |
|  |  | Sucralfate | 1.73 (1.43-2.08) | Ferrous sulfate | 1.71 (1.47-1.99) |
|  |  | CPS | 1.67 (1.41-1.98) | Famotidine | 1.69 (1.30-2.19) |
|  |  | Bismuth oxide | 1.66 (1.35-2.03) | Rabeprazole | 1.62 (1.26-2.08) |
|  |  | Diclofenac | 1.56 (1.28-1.89) | Tramadol | 1.60 (1.40-1.82) |
|  |  | Ferrous sulfate | 1.55 (1.33-1.80) | Trimebutine | 1.56 (1.29-1.88) |
|  |  | Ranitidine | 1.51 (1.31-1.75) | Tiropramide | 1.53 (1.24-1.89) |
|  |  | Cimetidine | 1.50 (1.28-1.77) | Simethicone | 1.49 (1.24-1.78) |
|  |  | Clopidogrel | 1.50 (1.23-1.82) | Chlorpheniramine | 1.48 (1.30-1.67) |
|  |  | Acetaminophen | 1.48 (1.30-1.68) | Ranitidine | 1.47 (1.28-1.69) |
|  |  | Chlorpheniramine | 1.46 (1.28-1.67) | Dihydrocodeine | 1.37 (1.18-1.58) |
|  |  | Dihydrocodeine | 1.41 (1.21-1.65) | Acetaminophen | 1.31 (1.16-1.48) |
|  |  |  |  | Aspirin | 1.30 (1.14-1.47) |

OR, odds ratio; CI, confidence interval; CPS, calcium polystyrene sulfonate.

| 2015 | | 2016 | | 2017 | |
| --- | --- | --- | --- | --- | --- |
| Medications | OR (95% CI) | Medications | OR (95% CI) | Medications | OR (95% CI) |
| Megestrol | 4.33 (2.27-8.63) | Haloperidol | 4.37 (2.24-8.93) | Oxycodone | 6.05 (3.30-11.74) |
| Valproate | 4.20 (2.15-8.63) | Megestrol | 4.32 (2.36-8.22) | Codeine | 3.10 (2.04-4.76) |
| Quetiapine | 4.13 (2.77-6.25) | Fentanyl | 3.80 (2.58-5.66) | Buprenorphine | 2.89 (1.72-4.94) |
| Bethanechol | 3.82 (2.40-6.19) | Rivaroxaban | 3.59 (1.92-6.96) | Megestrol | 2.83 (1.71-4.75) |
| Haloperidol | 3.74 (2.08-6.95) | Quetiapine | 3.58 (2.58-5.01) | Fentanyl | 2.68 (1.93-3.74) |
| Clonazepam | 3.49 (2.24-5.52) | Theophylline | 3.21 (1.75-6.06) | Quetiapine | 2.62 (1.98-3.49) |
| Levodopa | 3.14 (1.78-5.65) | Risperidone | 3.20 (1.79-5.84) | Triazolam | 2.48 (1.58-3.90) |
| Trazodone | 3.12 (1.98-4.98) | Memantine | 3.14 (1.83-5.51) | Pregabalin | 2.23 (1.66-3.00) |
| Lorazepam | 2.75 (2.00-3.81) | Buprenorphine | 2.97 (1.71-5.23) | Sodium bicarbonate | 2.15 (1.92-2.40) |
| Escitalopram | 2.59 (1.71-3.92) | Oxycodone | 2.93 (1.71-5.11) | Zolpidem | 2.07 (1.68-2.55) |
| Silodosin | 2.54 (1.60-4.07) | Lorazepam | 2.86 (2.08-3.95) | Ketorolac | 2.07 (1.48-2.88) |
| Fentanyl | 2.46 (1.65-3.68) | Mirabegron | 2.76 (1.73-4.45) | Trimetazidine | 2.03 (1.52-2.72) |
| Potassium chloride | 2.44 (2.05-2.92) | Clonazepam | 2.51 (1.75-3.61) | Nitroglycerin | 2.03 (1.51-2.73) |
| Solifenacin | 2.43 (1.57-3.79) | Solifenacin | 2.45 (1.62-3.74) | Alprazolam | 2.01 (1.60-2.53) |
| Propranolol | 2.31 (1.56-3.42) | Trazodone | 2.42 (1.62-3.61) | Clonazepam | 2.01 (1.43-2.82) |
| Celecoxib | 2.18 (1.58-3.02) | Amitriptyline | 2.41 (1.82-3.19) | Potassium chloride | 1.96 (1.67-2.29) |
| Alprazolam | 2.14 (1.69-2.72) | Bethanechol | 2.34 (1.56-3.54) | Lansoprazole | 1.91 (1.51-2.41) |
| Gabapentin | 2.12 (1.63-2.77) | Ketorolac | 2.32 (1.62-3.33) | Tamsulosin | 1.82 (1.53-2.17) |
| CPS | 2.10 (1.81-2.43) | Potassium chloride | 2.30 (1.94-2.72) | Pantoprazole | 1.81 (1.44-2.27) |
| Zolpidem | 2.10 (1.65-2.66) | Gabapentin | 2.29 (1.77-2.96) | Esomeprazole | 1.81 (1.51-2.17) |
| Sodium bicarbonate | 2.01 (1.79-2.26) | Isosorbide dinitrate | 2.26 (1.65-3.10) | Furosemide | 1.81 (1.60-2.04) |
| Furosemide | 1.97 (1.73-2.25) | Trimetazidine | 2.24 (1.67-3.02) | Dimenhydrinate | 1.80 (1.37-2.37) |
| Pregabalin | 1.97 (1.47-2.65) | Codeine | 2.19 (1.49-3.22) | Nicorandil | 1.74 (1.32-2.28) |
| Famotidine | 1.97 (1.53-2.53) | Pregabalin | 2.15 (1.59-2.92) | Diazepam | 1.68 (1.34-2.11) |
| Trimetazidine | 1.90 (1.37-2.62) | Cefpodoxime | 2.15 (1.49-3.09) | Torsemide | 1.66 (1.31-2.10) |
| Amitriptyline | 1.86 (1.40-2.48) | Lansoprazole | 2.13 (1.67-2.71) | Donepezil | 1.65 (1.31-2.07) |
| Trimebutine | 1.83 (1.52-2.21) | Nitroglycerin | 2.10 (1.56-2.83) | Gabapentin | 1.64 (1.27-2.11) |
| Donepezil | 1.82 (1.33-2.49) | Tamsulosin | 2.06 (1.71-2.47) | CPS | 1.62 (1.42-1.86) |
| Dimenhydrinate | 1.82 (1.35-2.46) | Sodium bicarbonate | 2.03 (1.82-2.27) | Diphenylpyraline | 1.62 (1.26-2.06) |
| Tramadol | 1.80 (1.58-2.04) | Furosemide | 2.02 (1.78-2.29) | Celecoxib | 1.61 (1.28-2.03) |
| Rabeprazole | 1.79 (1.40-2.29) | Zolpidem | 2.01 (1.62-2.50) | Rabeprazole | 1.57 (1.28-1.92) |
| Tamsulosin | 1.76 (1.45-2.15) | Nicorandil | 1.97 (1.50-2.60) | Diclofenac | 1.56 (1.30-1.88) |
| Torsemide | 1.73 (1.34-2.23) | Finasteride | 1.93 (1.42-2.61) | Simethicone | 1.54 (1.28-1.87) |
| Simethicone | 1.70 (1.42-2.02) | Famotidine | 1.88 (1.47-2.40) | Tramadol | 1.52 (1.35-1.70) |
| Diazepam | 1.67 (1.33-2.11) | Alprazolam | 1.78 (1.43-2.22) | Ranitidine | 1.51 (1.34-1.71) |
| Esomeprazole | 1.67 (1.34-2.07) | Celecoxib | 1.75 (1.37-2.23) | Trimebutine | 1.49 (1.22-1.82) |
| Spironolactone | 1.62 (1.27-2.07) | Simethicone | 1.72 (1.44-2.07) | Clopidogrel | 1.43 (1.23-1.67) |
| Levofloxacin | 1.61 (1.28-2.01) | Ferrous sulfate | 1.71 (1.49-1.97) | Chlorpheniramine | 1.42 (1.27-1.59) |
| Tiropramide | 1.58 (1.29-1.93) | Diclofenac | 1.70 (1.42-2.03) | Sucralfate | 1.39 (1.20-1.60) |
| Clopidogrel | 1.57 (1.33-1.86) | Nifedipine | 1.68 (1.37-2.06) | Bismuth oxide | 1.38 (1.19-1.60) |
| Ranitidine | 1.56 (1.37-1.78) | CPS | 1.67 (1.45-1.92) | Ferrous Sulfate | 1.37 (1.20-1.56) |
| Dimethylamine | 1.55 (1.27-1.89) | Tramadol | 1.67 (1.49-1.87) | Acetaminophen | 1.29 (1.16-1.44) |
| Acetaminophen | 1.48 (1.31-1.66) | Esomeprazole | 1.66 (1.38-2.00) | Aspirin | 1.28 (1.14-1.44) |
| Ferrous Sulfate | 1.42 (1.23-1.64) | Pantoprazole | 1.61 (1.27-2.04) |  |  |
| Cimetidine | 1.37 (1.17-1.59) | Rabeprazole | 1.59 (1.28-1.99) |  |  |
| Aspirin | 1.31 (1.16-1.48) | Diazepam | 1.58 (1.26-1.98) |  |  |
|  |  | Clopidogrel | 1.54 (1.32-1.80) |  |  |
|  |  | Tiropramide | 1.52 (1.25-1.85) |  |  |
|  |  | Trimebutine | 1.51 (1.23-1.85) |  |  |
|  |  | Dimethylamine | 1.47 (1.22-1.77) |  |  |
|  |  | Ranitidine | 1.41 (1.24-1.59) |  |  |
|  |  | Acetaminophen | 1.39 (1.25-1.55) |  |  |
|  |  | Carvedilol | 1.38 (1.18-1.61) |  |  |
|  |  | Aspirin | 1.37 (1.22-1.54) |  |  |
|  |  | Chlorpheniramine | 1.34 (1.20-1.50) |  |  |
|  |  | Dihydrocodeine | 1.32 (1.16-1.50) |  |  |


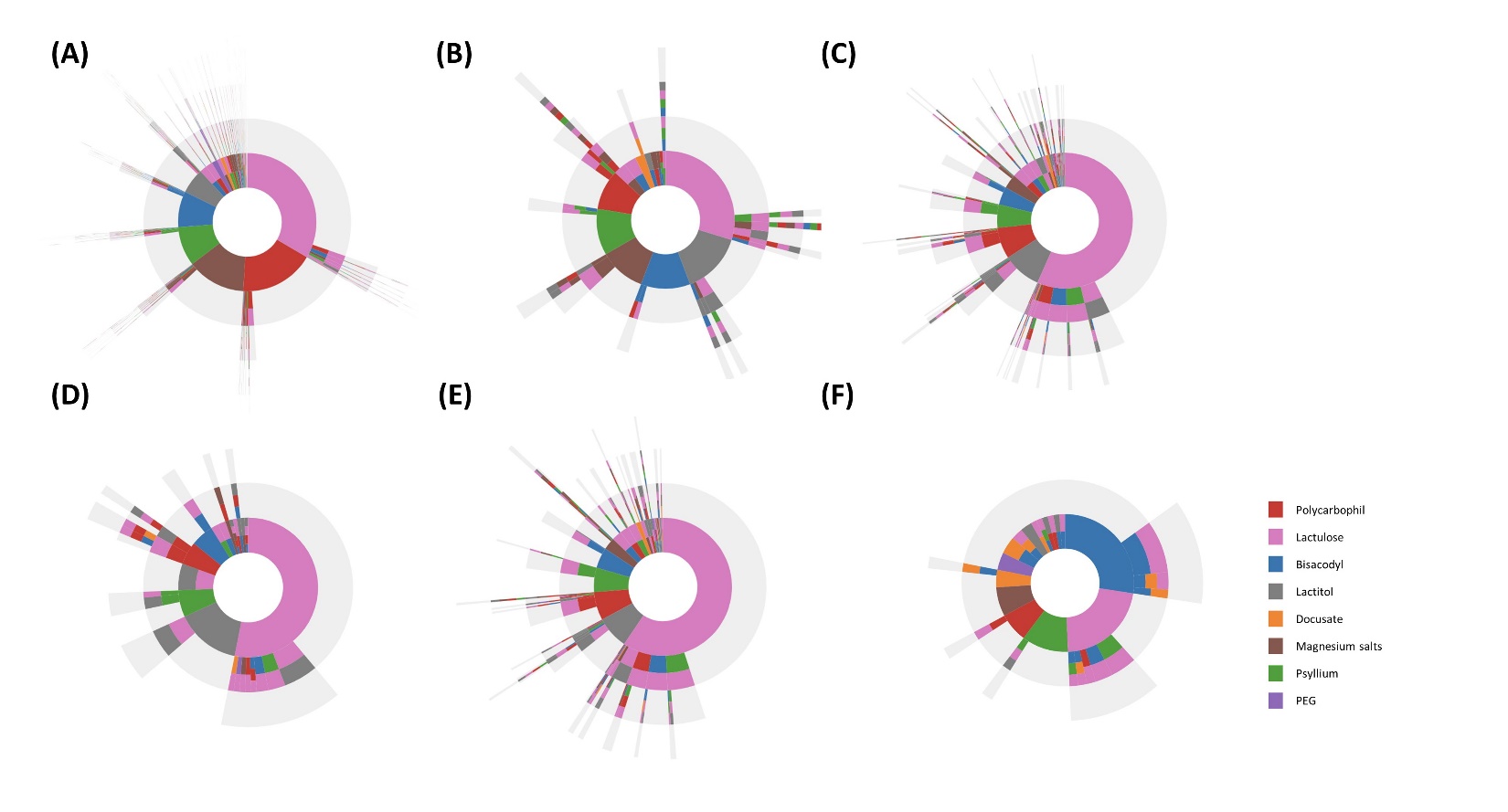


Supplementary Figure 1. Treatment pathway for laxatives in patients with (A) non-chronic kidney disease (non-CKD), (B) mild chronic kidney disease (CKD) (CKD stages 1-3), (C) advanced CKD (CKD stages 4-5), (D) peritoneal dialysis (PD), (E) hemodialysis (HD), and (F) kidney transplantation in 2012


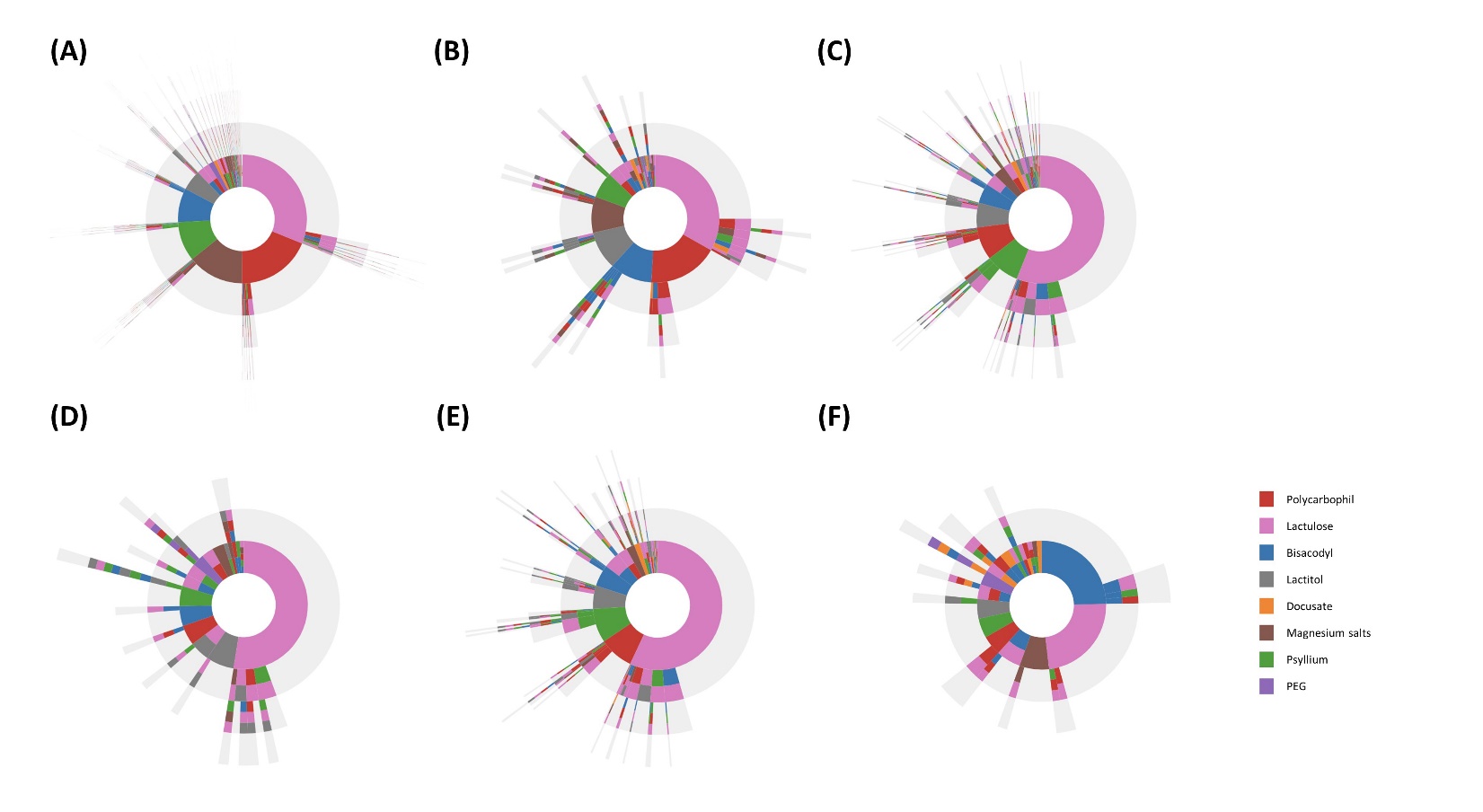


Supplementary Figure 2. Treatment pathway for laxatives in patients with (A) non-chronic kidney disease (non-CKD), (B) mild chronic kidney disease (CKD) (CKD stages 1-3), (C) advanced CKD (CKD stages 4-5), (D) peritoneal dialysis (PD), (E) hemodialysis (HD), and (F) kidney transplantation in 2013


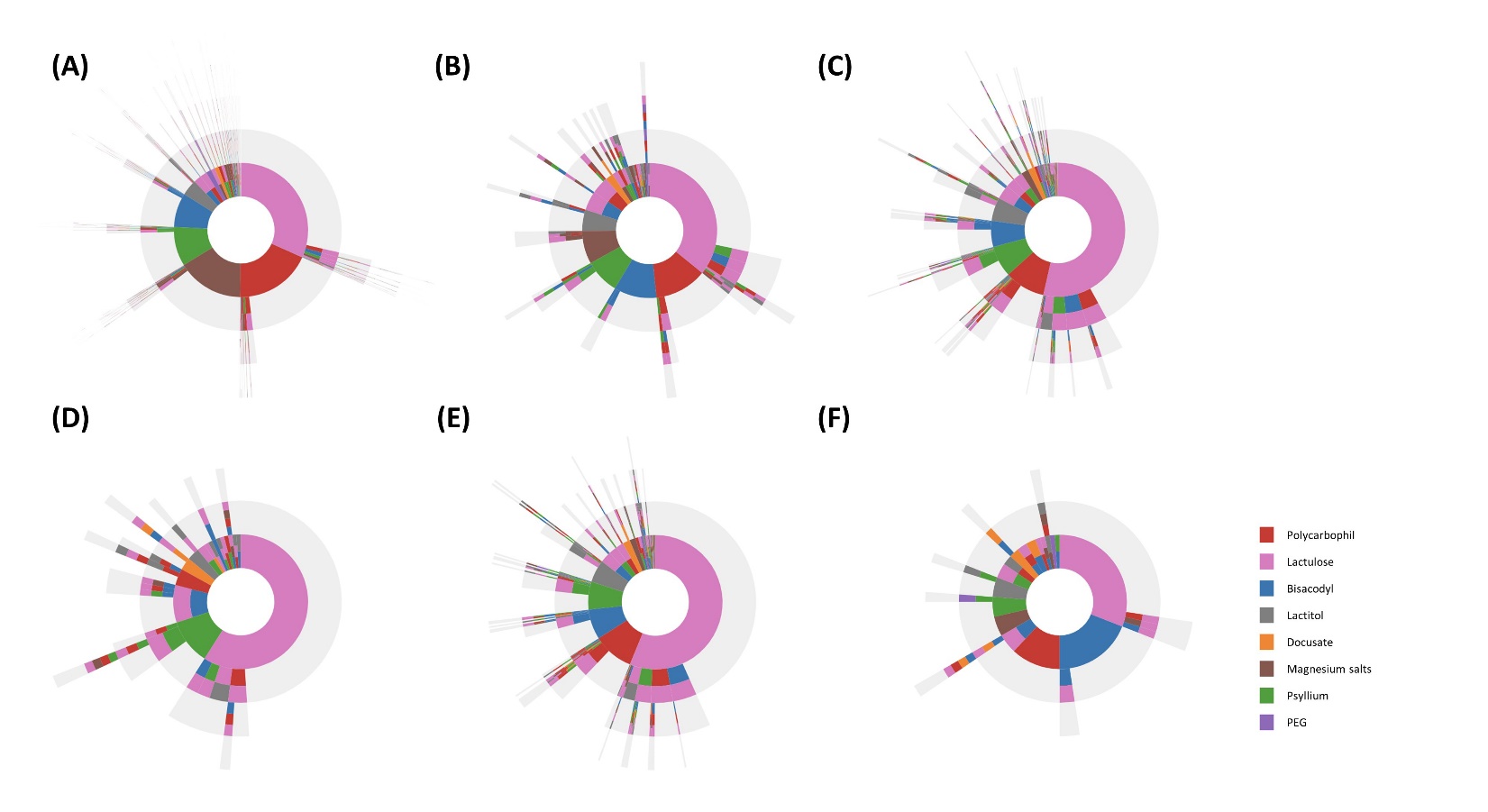


Supplementary Figure 3. Treatment pathway for laxatives in patients with (A) non-chronic kidney disease (non-CKD), (B) mild chronic kidney disease (CKD) (CKD stages 1-3), (C) advanced CKD (CKD stages 4-5), (D) peritoneal dialysis (PD), (E) hemodialysis (HD), and (F) kidney transplantation in 2014


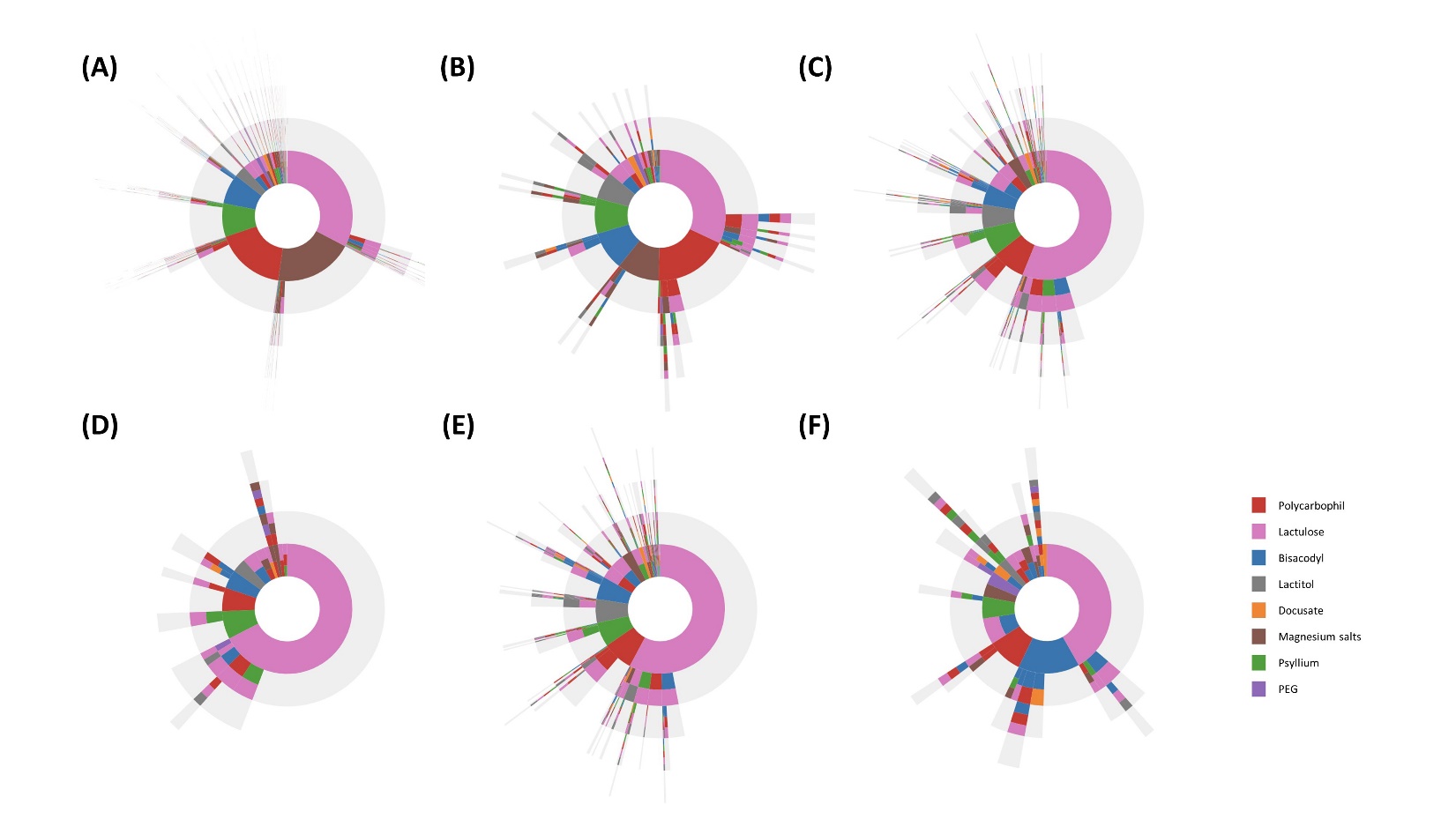


Supplementary Figure 4. Treatment pathway for laxatives in patients with (A) non-chronic kidney disease (non-CKD), (B) mild chronic kidney disease (CKD) (CKD stages 1-3), (C) advanced CKD (CKD stages 4-5), (D) peritoneal dialysis (PD), (E) hemodialysis (HD), and (F) kidney transplantation in 2015


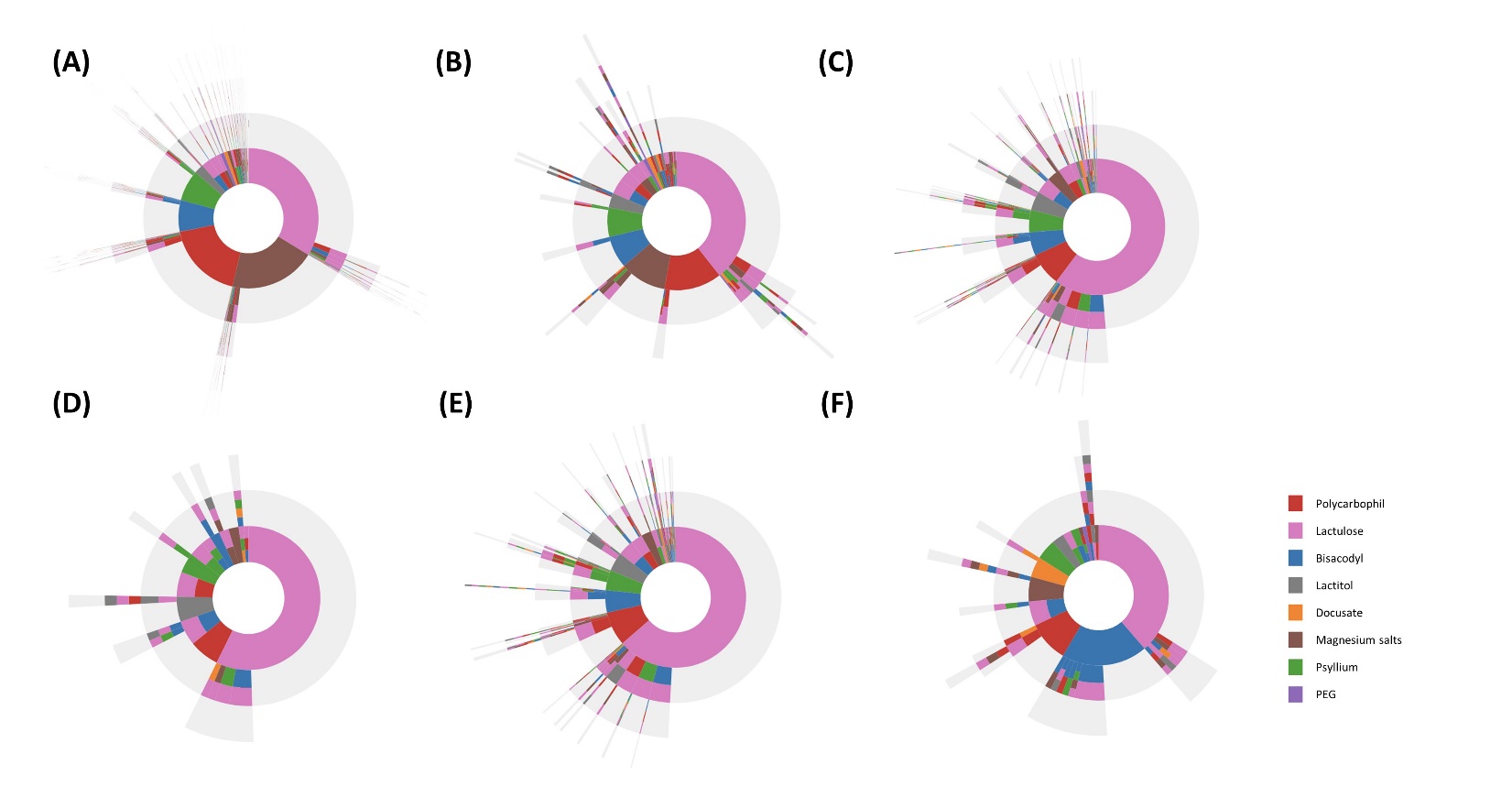


Supplementary Figure 5. Treatment pathway for laxatives in patients with (A) non-chronic kidney disease (non-CKD), (B) mild chronic kidney disease (CKD) (CKD stages 1-3), (C) advanced CKD (CKD stages 4-5), (D) peritoneal dialysis (PD), (E) hemodialysis (HD), and (F) kidney transplantation in 2016
